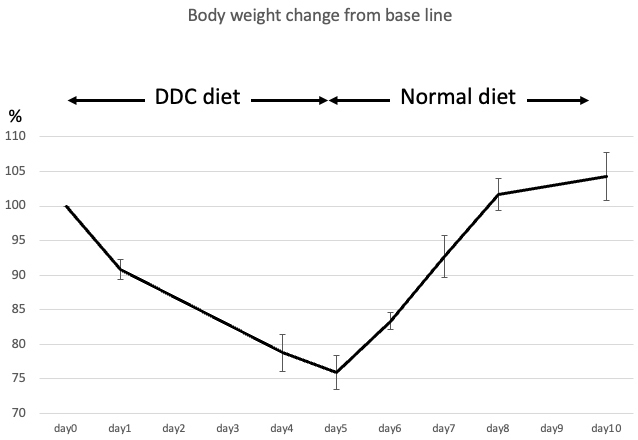# Mesenchymal stromal cell administration promotes macrophage-mediated bile duct regeneration

**DOI:** 10.1016/j.reth.2026.101065

**Published:** 2026-02-03

**Authors:** Takanori Igarashi, Naruhiro Kimura, Hiroyuki Abe, Tomoaki Yoshida, Yusuke Watanabe, Hiroteru Kamimura, Akira Sakamaki, Takeshi Yokoo, Kenya Kamimura, Atsunori Tsuchiya, Shuji Terai

**Affiliations:** a Division of Gastroenterology and Hepatology, Niigata University Graduate School of Medical and Dental Sciences, 1-757 Asahimachi-dori, Chuo-ku, Niigata, 951-8510, Japan; b Department of General Medicine, Niigata University Graduate School of Medical and Dental Sciences, 1-757 Asahimachi-dori, Chuo-ku, Niigata, 951-8510, Japan; c Department of Gastroenterology and Hepatology, Yamanashi University Graduate School of Medical and Dental Sciences, 1110 Shimokato, Chuo, Yamanashi, 409-3898, Japan

**Keywords:** Bile duct damage, Mesenchymal stromal cell therapy, Primary biliary cholangitis, Primary sclerosing cholangitis, Macrophage

## Abstract

**Introduction:**

Mesenchymal stromal cell (MSC) therapy is used in cirrhosis models of chronic hepatocellular damage and represents a novel therapeutic approach for liver regeneration. However, no studies have examined MSC therapy for bile duct injury and its effects on bile duct regeneration. This study aimed to elucidate the mechanism underlying bile duct regeneration by establishing a regeneration model and administering MSCs.

**Methods:**

A model of bile duct regeneration was developed by feeding mice a 3,5-diethoxycarbonyl-1,4-dihydrocollidine (DDC)-containing diet to induce bile duct injury, followed by a normal diet to promote bile duct regeneration. MSCs were administered concurrently with the normal diet, and the livers were analyzed after 2 days. An experimental system was also developed in which clodronate liposomes were administered alongside MSCs to eliminate the influence of macrophages.

**Results:**

MSC administration significantly increased the rate of body weight gain and the liver-to-body weight ratio on day 2 after the change to a normal diet. Immunostaining revealed an increase in the number of CK19^+^ cholangiocytes. The numbers of Hnf4α^+^ and Sox9^+^ cells were significantly elevated in the MSC-treated group. Flow cytometric analysis of liver lymphocytes showed a significant increase in the number of Ly6C^−^ macrophages in the MSC-treated group. Furthermore, analysis of liver lymphocyte populations and cap analysis of gene expression revealed alterations in the immune response and macrophage-related genes, whereas macrophage removal during MSC treatment abolished the bile duct regenerative effects observed with MSC treatment.

**Conclusions:**

Macrophages play a crucial role in bile duct regeneration, and MSC administration has regenerative effects. This approach has potential as a novel treatment modality for bile duct injuries.

## Abbreviations

CAGE: cap analysis of gene expression
Clips: clodronate liposomes
DEG: differentially expressed gene
DDC: 3,5-diethoxycarbonyl-1,4-dihydrocollidine
DR: ductular reaction
GO: Gene Ontology
IDR: irreproducible discovery rate
MSC: mesenchymal stem cell
NGP: neutrophilic granule protein
PBC: primary biliary cholangitis
PSC: primary sclerosing cholangitis

## 1 Introduction

Primary biliary cholangitis (PBC) and primary sclerosing cholangitis (PSC) are liver diseases characterized by the progressive destruction of the intrahepatic bile ducts. These diseases exhibit an autoimmune pathogenesis and may progress to liver cirrhosis and/or failure. Only a few treatments are available, including ursodeoxycholic acid [1,2], and liver transplantation remains the only option following progression to liver failure. Therefore, PBC and PSC are considered intractable, and novel methods to promote bile duct regeneration (BDR) are necessary for efficient liver regeneration. Bile duct cells constitute approximately 5 % of the liver [3]. However, incomplete regeneration of the bile duct tree results in incomplete liver regeneration [4]. Hence, in addition to the aforementioned regenerative treatments, novel BDR methods are essential for the treatment of bile duct-induced diseases.

The liver has the capacity to recover from diverse injuries, including bile duct damage, aging, fat accumulation, and certain diseases. However, prolonged or severe injury beyond its resilience can result in liver cirrhosis, likely leading to fatal complications, such as liver cancer and liver failure. Although cirrhosis may be reversible with antiviral treatment, it is generally an irreversible condition, requiring effective treatments for affected patients [[5], [6], [7]]. Watanabe et al. [8] reported that mesenchymal stem cell (MSC) injections during carbon tetrachloride-induced liver damage alleviated liver damage and prevented cirrhosis progression. MSCs exhibit both regenerative and immunomodulatory capabilities and have been used to treat autoimmune and inflammatory diseases, as well as injuries, in various animal and clinical studies [9].

MSCs are utilized in regenerative and other treatments across various fields, including neurology, cardiology, and hepatology, with several human trials currently underway. MSCs are positive for the common markers CD73, CD90, and CD105 and can differentiate into osteoblasts, adipocytes, and chondrocytes [10]. MSCs promote regeneration through anti-inflammatory and antifibrotic effects mediated by chemokines, cytokines, and exosomes. In the liver, these effects are particularly prominent, where MSCs function as “conducting cells” [11]. Importantly, MSCs generally exhibit low immunogenicity, express low or modest levels of major histocompatibility complex class I molecules, and lack the expression of major histocompatibility complex class II and co-stimulatory molecules. Although human-to-mouse transplantation poses challenges for basic research, xenograft models using MSCs have emerged as valuable tools [12] to enhance our understanding of their therapeutic mechanisms. Recent experimental and clinical studies in cholangiopathies, including PBC have shown that MSCs exert immunosuppressive and cytoprotective effects within the biliary compartment, alleviating portal inflammation and bile duct injury [13]. MSCs modulate immune responses and promote macrophage polarization toward reparative phenotypes through paracrine mediators and extracellular vesicles [14], while macrophages themselves are key regulators of ductular reaction (DR), matrix remodeling, and biliary epithelial repair [15]. In this context, we hypothesized that MSC administration promotes bile duct regeneration by modulating macrophage phenotype and activity within the injured biliary microenvironment, thereby facilitating cholangiocyte proliferation and bile duct reconstruction.

In addition to their well-established hepatoprotective effects, MSCs release cytokines, chemokines, and extracellular vesicles that modulate immune responses within the biliary microenvironment. These paracrine signals can promote macrophage polarization toward a reparative phenotype that supports cholangiocyte proliferation and bile duct remodeling. Therefore, this study aimed to determine whether MSC administration promotes bile duct regeneration through macrophage-mediated mechanisms in a murine model of bile duct injury.

## 2 Methods

### 2.1 Animals

C57BL/6 male mice were purchased from Charles River (Yokohama, Japan). Mice were housed in a specific pathogen-free environment and kept under standard conditions with a 12-h day/night cycle and *ad libitum* access to food and water. All animal experiments were performed in compliance with the regulations and approved by the Institutional Animal Care and Use Committee at Niigata University. Body weights were scaled every day, and the liver/body weight ratio was measured at the time of harvest.

### 2.2 Cell transplantation

Human adipose tissue-derived MSCs (passage 2) were obtained from PromoCell (cat#: C-12977, Heidelberg, Germany) and expanded until passage 4 using StemPro MSC SFM XenoFree medium (Thermo Fisher Scientific, Waltham, MA, USA) at 37 °C.

### 2.3 Mouse model of BDR

The mice were fed a 3,5-diethoxycarbonyl-1,4-dihydrocollidine (DDC) diet for 5 days. The food was changed to a normal diet on day 5 to initiate regeneration. At the start of the regeneration period (day 5), 1 × 10^6^ MSCs (MSC group) or the same amount of PBS (BDR group) was injected through the tail vein. Liver and serum were collected from the mice on day 7 (DDC diet for 5 days + normal diet for 2 days). As in the aforementioned model, clodronate liposomes (Clips; FUJIFILM Wako, Japan) were administered intraperitoneally on the same day as the MSCs after a 5-day DDC diet. After 2 days of BDR on a normal diet, liver and serum were obtained and analyzed as the MSC + Clip group.

### 2.4 Immunostaining and immunofluorescence

For immunohistochemistry, 10 % formalin-fixed liver tissue was sliced into 4 μm-thick sections. Heat-mediated antigen retrieval was performed in 10 mM sodium citrate buffer (pH 6.0) when necessary. Primary antibodies (Supplemental Table 1) were incubated overnight at 4 °C. The slides were then stained using a second antibody, followed by incubation with a donkey anti-rabbit secondary antibody (Alexa Fluor Plus 594, A48254, Thermo Fisher Scientific; 1:400) and a goat anti-rat secondary antibody (Alexa Fluor 594, Thermo Fisher Scientific; 1:400) and mounted using VECTASHIELD mounting medium with DAPI (Vector Laboratories).

Images were acquired using an HS All-in-One Fluorescence Microscope (BZ-9000; Keyence, Osaka, Japan). The numbers of positive and negative cells in 10 randomized fields were counted and averaged.

### 2.5 Flow cytometry analyses

Liver tissues were chopped and passed through a 70-μm mesh (Greiner Bio-One). The supernatant was spun at 700 g for 20 min with 33 % Percoll and aspirated. Lymphocytes were pelleted via centrifugation at 400*g* for 5 min, and cells were resuspended in 2 % FBS-supplemented PBS. Single-cell suspensions were analyzed using the Zombie NIR Cell Viability Kit (BioLegend, San Diego, CA, USA) and washed with PBS. Cells were then incubated with the respective antibody cocktail at appropriate concentrations and reconstituted in PBS containing 2 % fetal calf serum. The monoclonal antibodies used for flow cytometry are listed in Supplemental Table 2. Raw data were obtained using a CA3800 cell analyzer (Sony, Tokyo, Japan) and analyzed using the FlowJo software.

### 2.6 Cap analysis of gene expression (CAGE) and computational analysis

CAGE library preparation, sequencing, mapping, gene expression analysis, motif discovery analysis, and Gene Ontology (GO) enrichment analysis were performed using DNAFORM (Yokohama, Kanagawa, Japan). Immune cells from the liver were isolated using methods similar to those employed in flow cytometry, and CD45^+^ cells were sorted using MojoSort beads (BioLegend).

Nascent RNA was extracted from cells using a previously described method. cDNA was synthesized from nascent RNA using random primers. The ribose diols in the 5ʹ cap structures of RNAs were oxidized and biotinylated. Biotinylated RNA/cDNA was selected using streptavidin beads (cap-trapping). Double-stranded cDNA libraries (CAGE libraries) were constructed following RNA digestion using RNase ONE/H and adaptor ligation to both ends of the cDNA.

The CAGE libraries were sequenced using single-end reads of 75 nucleotides on a NextSeq 500 instrument (Illumina). The V8 obtained reads (CAGE tags) were mapped to the GRCm39 genome using STAR software (version 2.7.9a).

CAGE tag clustering, identification of differentially expressed genes (DEGs), and motif discovery were performed using the RECLU pipeline [16]. Tag count data were clustered using a modified Paraclu program. Clusters with <0.1 counts per million were discarded. Regions with a 90 % overlap between replicates were extracted using BEDtools (version 2.12.0). Clusters with an irreproducible discovery rate (IDR) ≥ 0.1 and clusters longer than 200 bp were discarded. DEGs were identified using the edgeR package (version 3.22.5). For motif analysis, the genomic DNA sequence of the region from 200 bp upstream to 50 bp downstream of the differentially expressed CAGE peaks was subjected to the *de novo* motif discovery tools AMD, GLAM2, DREME, and Weeder. The presence of these motifs was examined with FIMO. The similarity between the consensus motifs and motifs in the JASPAR2020 CORE vertebrate database was evaluated using Tomtom. DEGs identified using RECLU with a false discovery rate ≤0.05 were used for GO enrichment analysis using the clusterProfiler package [17].

### 2.7 Statistical analysis

Statistical analyses were performed using GraphPad Prism 5 software (GraphPad Software Inc., La Jolla, CA, USA) and R software (Microsoft, Redmond, WA, USA). Data are presented as means ± standard deviations; all data were normally distributed. The results were assessed using the Mann–Whitney *U* test, the Kruskal–Wallis test followed by the Steel–Dwass post-hoc test, or Spearman's rank correlation test, as appropriate. Statistical significance was set at p < 0.05. A description of the experimental procedures is provided in the Supporting Information.

## 3 Results

### 3.1 Characteristics of BDR process after DDC diet

**Fig. 1:**
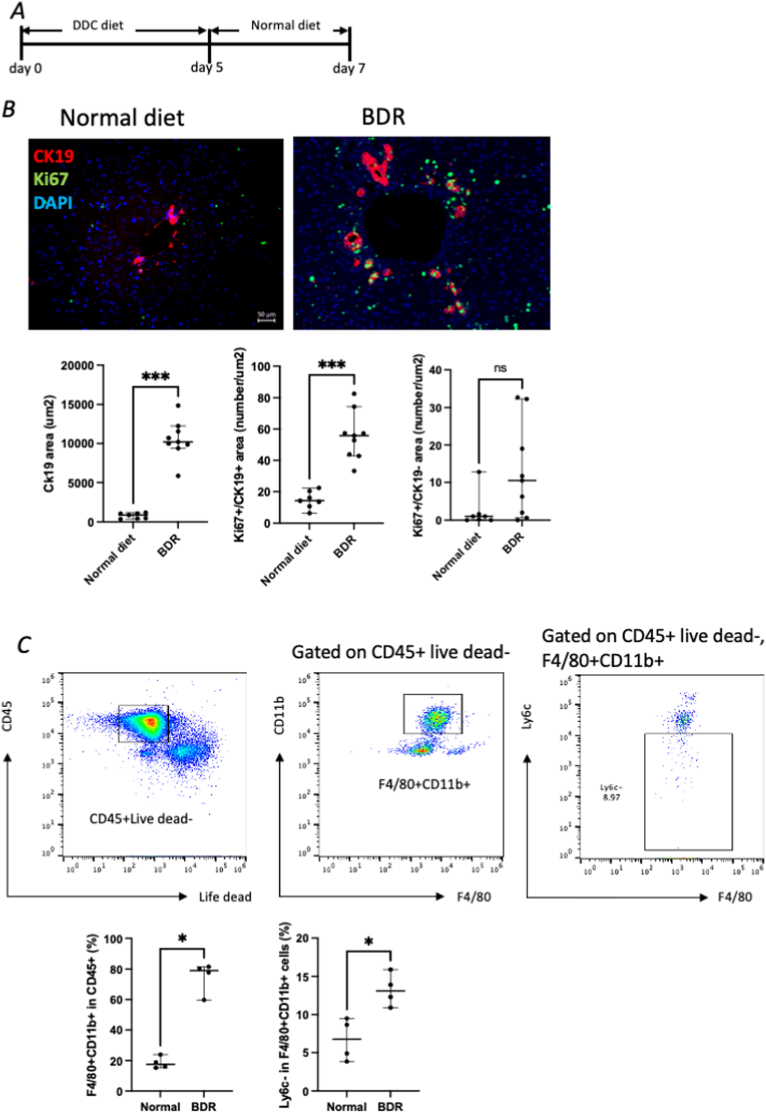
Characteristics of bile duct regeneration process after DDC diet (A) Schematic overview of the DDC-induced cholestatic injury protocol. (B–D) Histological and immunofluorescence analyses demonstrate a pronounced ductular reaction, characterized by the expansion of CK19^+^ cholangiocytes and increased Sox9 expression, confirming the successful induction of bile duct injury. This model served as the basis for evaluating MSC-associated regenerative effects.

Initially, the DDC diet was administered for 5 days; however, as body weight improved to the same level as before the DDC diet 2 days after the change to the normal diet (Supplemental Fig. 1), a further study was conducted on day 2 after the change to the normal diet. The schema of the BDR model is illustrated in Fig. 1A. CK19^+^ areas were significantly increased in the BDR model (10201 ± 2416 μm^2^) compared with the normal diet (873 ± 347 μm^2^; p < 0.001) and DDC 7 days mice (1291 ± 505 μm^2^; p = 0.039) (Fig. 1B). The number of Ki67^+^ cells among CK19^+^ cells was significantly increased in both BDR mice (55.8 ± 15.4) and DDC 7 days mice (40.4 ± 26.9) compared with normal diet mice (14.2 ± 5.4; p = 0.001 and p = 0.021, respectively). Ki67^+^ cells among CK19^-^ cells did not differ significantly among groups (normal diet, 1 ± 4; DDC 7 days mice, 18 ± 10; BDR mice, 10 ± 12). The immune cell changes after a specific diet were analyzed with flow cytometry (Fig. 1C). The percentage of F4/80^+^ CD11b^+^ macrophages in BDR mice (79 ± 10 %) was significantly higher than that in the normal diet mice (17 ± 4 %; p = 0.024). The percentage of Ly6c^−^ cells in the F4/80^+^ CD11b^+^ cells in BDR mice (13 ± 2 %) was significantly higher than that in the DDC 7 days mice (2 ± 1 %; p = 0.0071).

### 3.2 MSC treatment increased scar-healing macrophages during BDR

**Fig. 2:**
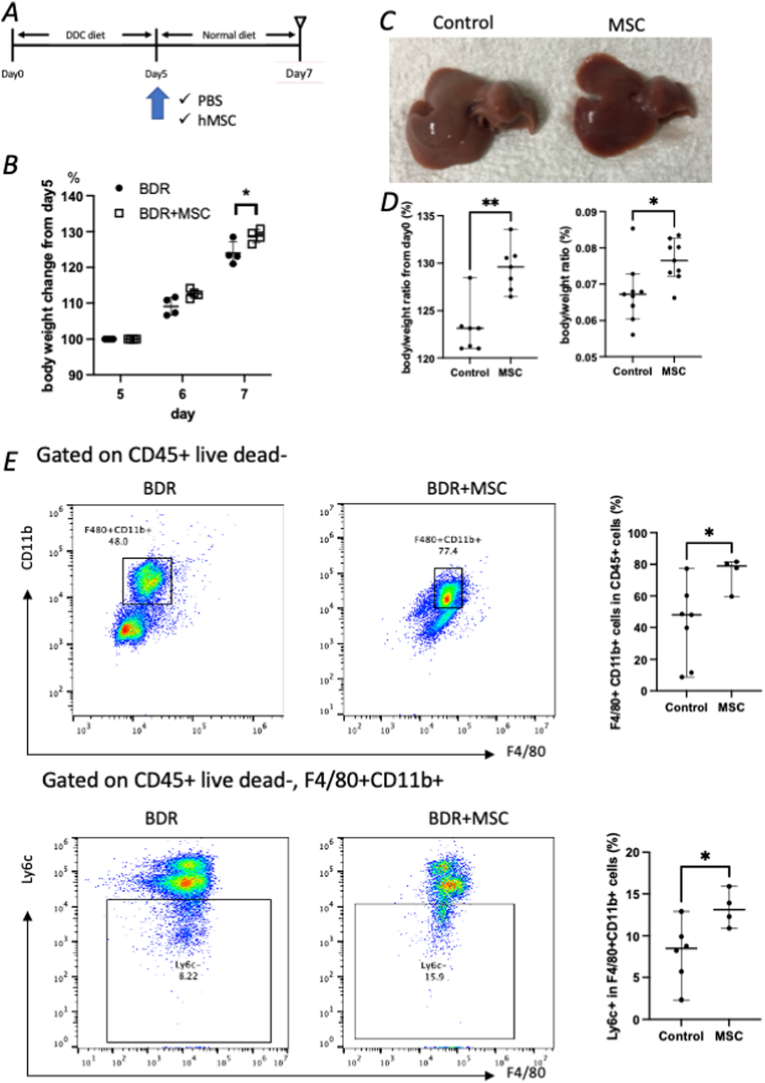
MSC treatment increases scar-healing macrophages during bile duct regeneration (A) Experimental outline for MSC treatment. (B–D) Histological and immunofluorescence images show broader CK19^+^ ductular expansion in mice receiving MSCs compared to controls. (E–G) MSC administration is associated with increased populations of Sox9^+^ and Ki67^+^ regenerative epithelial cells, indicating an enhanced biliary repair response.

MSCs (1 × 10^6^) were injected when the normal diet was initiated (Fig. 2A). The body weight change from baseline (at the start of the DDC diet) in the MSC group was significantly increased compared with that in the control group (129.6 ± 2.4 % vs. 123.1 ± 2.6 %, p = 0.004) (Fig. 2B). The liver/body weight ratio was also significantly increased in the MSC group (0.076 ± 0.006 vs. 0.067 ± 0.008, p = 0.002) (Fig. 2D), suggesting that MSC treatment accelerated the recovery from bile duct damage. Flow cytometry revealed a significantly increased percentage of macrophages in the MSC group (74.8 ± 10.2 % vs. 42.0 ± 24.8 %, p = 0.024). Among these, the proportion of Ly6C^−^ macrophages, which are the scar-healing macrophages, was significantly increased in the MSC group (13.3 ± 2.2 % vs. 7.9 ± 3.6 %, p = 0.038) (Fig. 2E). These data suggest that MSC treatment accelerated BDR by activating scar-healing macrophages.

### 3.3 MSC treatment increased ductular reaction (DR) by increasing Sox9^+^ hepatocytes

**Fig. 3:**
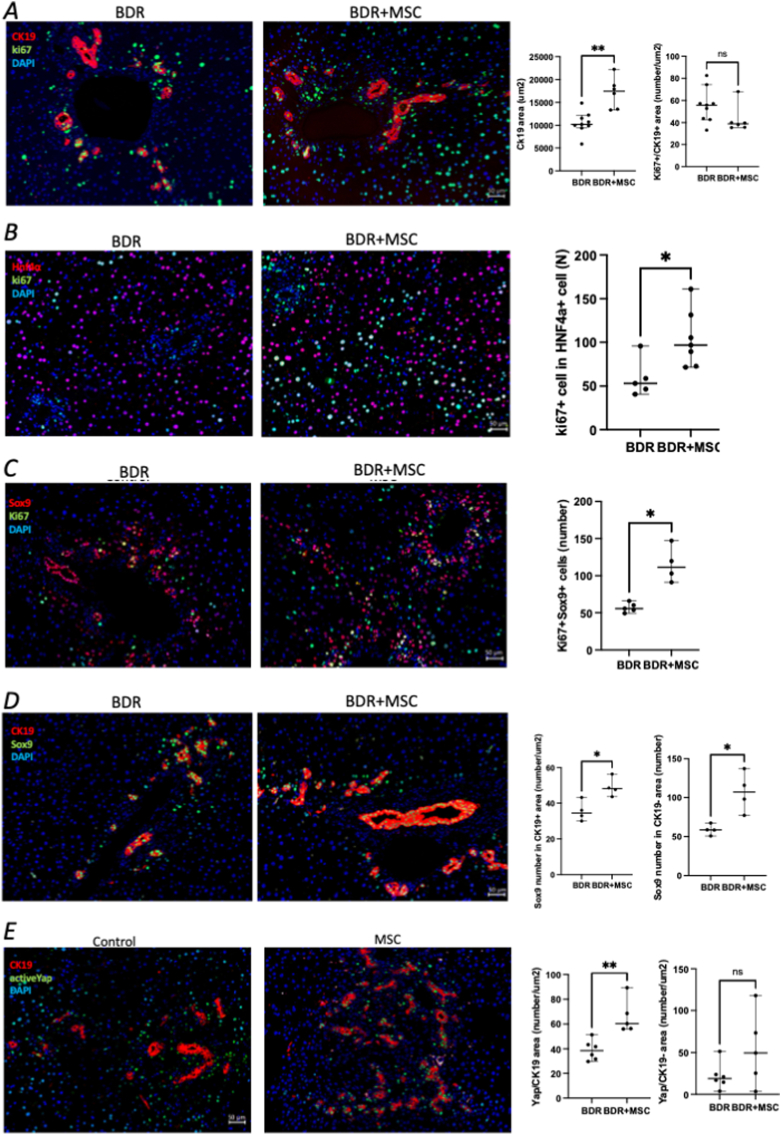
MSC treatment increases ductular reaction by increasing Sox9^+^ hepatocytes. (A) Representative images of active YAP staining within CK19^+^ areas. (B–D) MSC-treated mice display a stronger YAP^+^ signal in biliary epithelial regions compared with controls. (E) Quantification demonstrates an MSC-associated increase in YAP activation, consistent with the engagement of YAP-dependent regenerative pathways.

Immunofluorescence analysis showed significantly increased areas of CK19^+^ cells, indicating DR in the MSC group compared with the control group (17454 ± 3358 μm^2^ vs. 10201 ± 2416 μm^2^; p = 0.002) (Fig. 3A). However, the number of Ki67^+^ cells in the CK19^+^ population in the MSC group did not increase (Fig. 3A). Instead of bile duct cells, Ki67^+^ cells were increased in the Hnf4α^+^ population, and Ki67^+^ Hnaf4α^+^ cells were significantly increased in the MSC group compared with the control group (96.8 ± 32.4 vs. 53.0 ± 21.7; p = 0.018) (Fig. 3B). These Ki67^+^ cells were also Sox9^+^, which is a liver stem cell marker, and Ki67^+^/Sox9^+^ cells were significantly increased in the MSC group compared with the control group (111.4 ± 24.2 vs. 55.6 ± 6.5; p = 0.016) (Fig. 3C). These Sox9^+^ cells were also increased in the CK19^+^ populations after MSC administration (35.6 ± 5.7 vs. 49.1 ± 5.4; p = 0.029) (Fig. 3D). The Sox9^+^/CK19^-^ cells, which were considered Hnf4α^+^ hepatocytes, were also increased in the MSC group compared with the control group (107.0 ± 25.7 vs. 58.6 ± 6.8; p = 0.029) (Fig. 3D). These data suggest. that MSC treatment increased the number of Sox9^+^/Hnf4α^+^ cells, indicating the activation of bipotent progenitors or transitional hepatocytes that contribute to the DR, rather than a direct hepatocyte-to-cholangiocyte conversion. Active YAP^+^ cells were predominantly localized within CK19^+^ ductular structures, and their number was significantly higher in the MSC-treated group (60.4 ± 13.9 vs. 38.5 ± 8.1; p = 0.004). This indicates that MSC administration enhances Yes-associated protein (YAP) activation in proliferating cholangiocytes, suggesting a potential role for YAP signaling in MSC-induced bile duct regeneration.

### 3.4 Macrophage-related genes were significantly related in CAGE sequencing

**Fig. 4:**
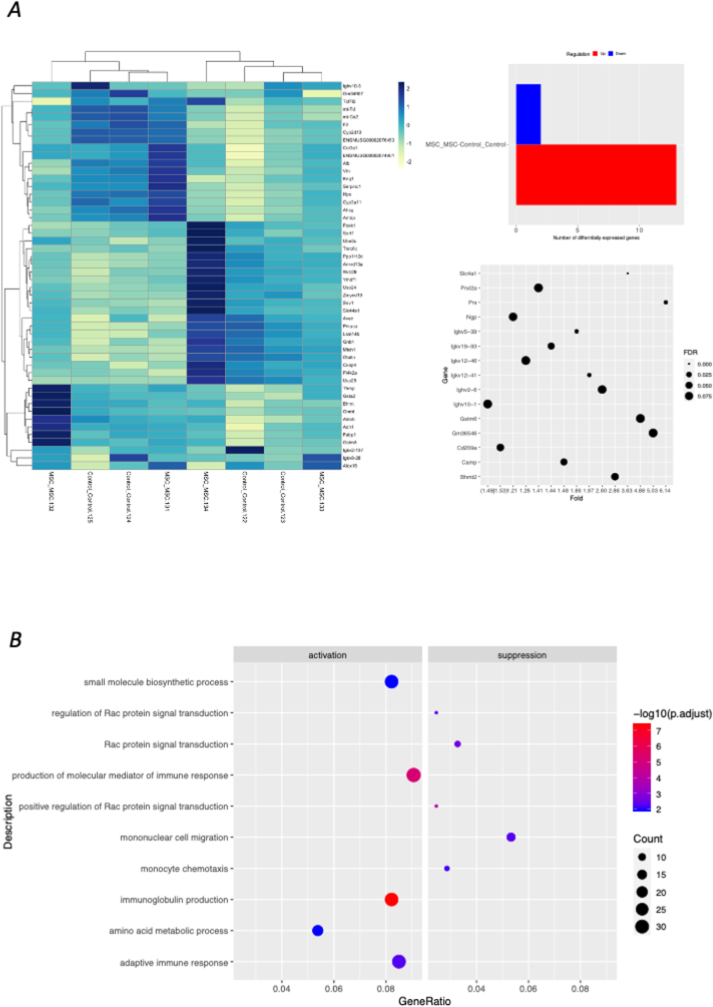
Macrophage-related genes are significantly associated with MSC-induced changes in cap analysis of gene expression (CAGE) sequencing analysis (A) Heat map displaying global gene expression patterns obtained from CAGE analysis, demonstrating distinct transcriptional signatures between control and MSC-treated groups. (B–C) Differential expression analysis highlights gene clusters altered by MSC treatment, including the downregulation of inflammatory monocyte-related genes and Rac-signaling-associated pathways. (D) Gene Ontology enrichment analysis shows that MSC administration is associated with reduced inflammatory and chemotactic processes, as well as transcriptional shifts consistent with a more reparative tissue environment. Genes emphasized in the main text (e.g., *Gstm6*, *Camp*, *Ngp*, *Cd209a*) are annotated in the figure.

To investigate the influence of MSCs on immune cells during BDR, CAGE sequencing of CD45^+^ cells from the liver was performed. To visualize the global transcriptional differences between the control and MSC-treated groups, we generated a heat map using the top 50 differentially expressed genes ranked by adjusted p-value. This selection was made to avoid excessive crowding of labels while retaining the most informative gene set. Gene symbols for all 50 genes are clearly displayed on the y-axis, allowing for direct confirmation of their expression patterns. Importantly, genes such as *Gstm6*, *Camp*, *Ngp*, and *Cd209a*, which were listed in the DEG analysis, are included in this top-50 set and are readily identifiable in the figure. The enrichment analysis heat map showed the gene trends, and DEG analysis revealed 15 genes with significant between-group differences. In addition to regeneration-related genes, including *Gstm6*, *Camp*, and *Ngp*, macrophage-related genes, such as *CD209a*, were also significantly altered (Fig. 4A). GO analysis revealed that MSC treatment significantly decreased the expression of Rac-, mononuclear cell-, and monocyte-related genes, suggesting the role of macrophages (Fig. 4B).

### 3.5 Macrophage depletion negated MSC treatment-induced improvements in BDR

**Fig. 5:**
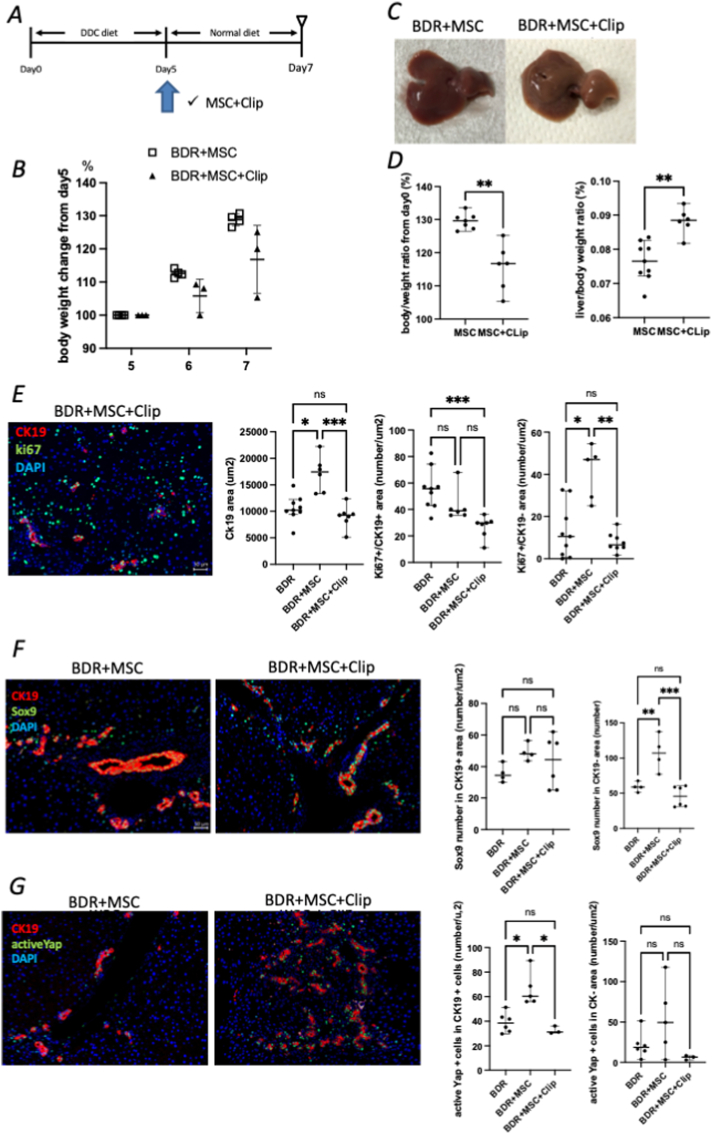
Macrophage depletion negates MSC treatment-induced improvements in bile duct regeneration (A) Schematic illustration of the experimental design, including MSC administration and clodronate liposome-mediated macrophage depletion. (B–D) Clodronate treatment in combination with MSCs resulted in marked body weight loss compared with MSC treatment alone. (E) Immunofluorescence imaging demonstrated that the depletion of macrophages attenuated MSC-associated bile duct regeneration, as shown by a reduction in CK19^+^ ductular structures and decreased Ki67^+^ proliferating cells within CK19^-^ regions. (F) Sox9^+^ cells within the CK19^-^ compartment were also diminished in the MSC + Clip group. (G) Active YAP^+^ cells within CK19^+^ biliary areas were reduced following macrophage depletion, indicating that macrophages contribute to the MSC-associated enhancement of biliary regenerative signaling.

Clip injections with MSCs were administered to investigate the importance of macrophages during BDR (Fig. 5A). The body weight change from baseline was significantly reduced in the MSC + Clip group (116.7 ± 7.1 vs. 129.6 ± 2.4; p = 0.001) (Fig. 5B). However, the liver/body weight ratio was significantly increased in the MSC + Clip group, suggesting that the body weight loss was strongly affected (Fig. 5D).

Immunofluorescence analysis showed that the CK19^+^ cell area was significantly reduced in the MSC+Clip group compared with the MSC group (9261 ± 2137 μm^2^, 17454 ± 3358 μm^2^; p = 0.001) (Fig. 5E). Furthermore, the number of Ki67^+^ cells in the CK19^-^ area was significantly decreased in the MSC + Clip group (6.6 ± 4.5 vs. 47.0 ± 12.9; p = 0.002) (Fig. 5E). The number of Sox9^+^ cells in the CK19^-^ population was significantly decreased in the MSC + Clip group compared with the MSC group (57.2 ± 15.2 vs. 107.0 ± 25.7; p = 0.009) (Fig. 5F). Additionally, the number of active Yap^+^ cells in the CK19^+^ population was significantly decreased in the MSC+Clip group compared with the MSC group (31.2 ± 3.1 vs. 60.4 ± 13.9; p = 0.002) (Fig. 5G).

## 4 Discussion

In this study, a BDR model was developed, and the effect of MSC administration on BDR was examined. Furthermore, the bile duct regenerative effect of MSCs was abolished by macrophage depletion, indicating the importance of macrophages in BDR.

Cholangiocytes transport bile produced by hepatocytes to the intestinal tract, forming a key component of the liver's homeostatic gradient [18]. Cholangiocytes constitute only 5 % of the liver [3], but liver regeneration remains incomplete when the bile duct tree is not fully regenerated and the bile duct epithelium is dysfunctional [4]. DR is a common response to bile duct damage caused by autoimmune liver diseases, such as PBC and PSC, as well as drugs, and also reflects a response to bile duct cell regeneration [19]. DR cells differentiate from liver progenitor cells [20]. Notably, Sox9 is a stem cell marker associated with DR [21], and EpCAM^–^/Sox9^+^ cells sorted and cultured in three-dimensional systems exhibit a biphenotypic potential, enabling their differentiation into hepatocytes and cholangiocytes [22]. In this study, although Sox9 was expressed in the bile duct, it was more abundantly expressed in CK19^–^ cells, specifically in Hnf4α^+^ hepatocytes, and was also co-expressed with Ki67.

YAP is a key effector of the Hippo pathway and has been shown to regulate cholangiocyte proliferation and DR during biliary repair [23,24]. YAP is believed to be essential for cholangiocyte proliferation, whereas DR is essential in some injury models, such as bile duct ligation and CCL4-induced liver injury [20,21,23,24]. The increase in active YAP^+^ cells within CK19^+^ areas following MSC administration suggests that MSC-derived paracrine signaling or macrophage-mediated cues may enhance YAP activation in biliary epithelial cells, thereby promoting ductular expansion and bile duct regeneration. Therefore, this result suggests that MSC administration promotes BDR in DDC-induced bile duct injury.

Macrophages play an important role in liver regeneration. Watanabe et al. [8] showed that tail-injected MSCs are mostly trapped in the lungs and exert their effect at injury sites via extracellular vesicles [8]. MSCs change the polarity of macrophages towards an anti-inflammatory phenotype, increase the production of matrix metalloproteinases to reduce the extracellular matrix, and increase the phagocytosis of hepatocyte debris, during which macrophages upregulate pro-regenerative factors [8]. Therefore, MSCs can act as “conduction cells” and indirectly control immune cells. In this study, MSC administration increased the number of Ly6C^−^ macrophages. Ly6C^lo^ macrophages have attracted wide attention for their protective roles in wound healing, anti-inflammatory processes, and antifibrotic processes [[25], [26], [27]]. Several genes associated with inflammatory monocyte and Rac-related signaling pathways, including *Gstm6*, *Camp*, *Ngp*, and *Cd209a*, were downregulated in MSC-treated mice. These transcriptional changes are consistent with the attenuation of Ly6C^+^ inflammatory monocyte/macrophage activity rather than a global activation of macrophages. Along with the increased proportion of Ly6C^−^ repair-associated macrophages, the data suggest that MSC administration modulates hepatic macrophage populations toward a reparative phenotype. This macrophage reprogramming provides a plausible mechanism linking MSC treatment to the enhanced bile duct regeneration observed in this study. Notably, colony-stimulating factor 1 promotes the maturation of macrophages from bone marrow-derived macrophage precursors, accompanied by a rapid decrease in Ly6C expression [28,29]. Furthermore, IL-4 and IL-10 can promote the conversion of liver-derived Ly6C^hi^ macrophages into Ly6C^lo^ macrophages, with a synergistic effect between the two cytokines [30]. Hepatic macrophages have been reported to support biliary repair through cytokines such as IL-10 and TGF-β, as well as through matrix remodeling enzymes, by localizing to the periductular niche where they modulate cholangiocyte proliferation [15,31,32]. Loss of these macrophage-derived signals through clodronate depletion may therefore reduce the cytokine and extracellular matrix environment that promotes the expansion of Sox9^+^ and CK19^+^ biliary epithelial cells. Although we did not directly measure these mediators or assess macrophage–duct interactions, our findings are consistent with a model in which MSC treatment enhances bile duct regeneration partly through the preservation or reprogramming of such pro-repair macrophage populations. The reduction of MSC-associated regenerative responses in macrophage-depleted mice aligns with macrophage involvement, although non-macrophage-specific effects of clodronate cannot be excluded. To our knowledge, this is the first study to investigate the effects of MSC administration on Ly6C expression in macrophages. Although detailed studies of the bile ducts have not been possible, some MSCs may promote the differentiation of hepatocytes from Sox9^+^ biphenotypic cells into cholangiocytes. Further work will be needed to clarify whether MSC-associated signals directly influence the lineage allocation of Sox9^+^ biphenotypic cells.

Treatments in humans have also been investigated. In a Phase II study of patients with alcoholic cirrhosis, liver biopsy results at 6 months after treatment showed a 25 % reduction in the fibrosis area after one dose and a 37 % reduction after two doses, along with an improvement in Child–Pugh scores [33]. Moreover, treatment of patients with hepatitis B-related acute-on-chronic liver failure may improve total bilirubin levels and end-stage liver disease scores [34]. This study can serve as the basis for further research and clinical trials on cholangiopathic diseases in humans, with the potential to reduce the progression to liver transplantation and mortality from liver failure.

This study also has some limitations. First, we used a drug-induced bile duct injury mouse model with a DDC diet. Thus, whether MSC administration in other cholestatic liver injury models, such as bile duct ligation, accelerates BDR or not remains unknown. Second, although we demonstrated the usefulness of MSC administration in an acute bile duct injury model, we did not demonstrate this in a chronic bile duct injury model. Hence, the usefulness of MSC administration in the chronic bile duct injury model is unclear. Third, we did not examine the side effects associated with MSC administration.

## 5 Conclusions

The effect of MSC administration on BDR was mediated by Ly6C^−^ macrophages and was negated by macrophage depletion using Clips. Specifically, Ly6C^−^ macrophages promote hepatocyte differentiation from Sox9^+^ cells to bile duct cells. MSC therapy, which is already used in humans, may be applied to bile duct disorders in the future to improve the poor prognosis of these diseases.

## Ethical approval

All animal experiments were performed in compliance with the institutional regulations, and the study protocols were approved by the Institutional Animal Care and Use Committee of Niigata University.

## Consent for publication

Not applicable.

## Authors contributions

T.I. and N.K. collected and analyzed the data and wrote the manuscript. H.A., T.Y., Y.W., H.K., A.S., T.Y., K.K., A.T., and S.T. collected and analyzed the data. S.T. supervised the manuscript and project. All authors reviewed the manuscript. All authors read and approved the final manuscript.

## Availability of data and materials

All data required to evaluate the conclusions of this study are presented in the paper and supplementary materials. Additional data related to this study may be requested from the authors.

## Funding

This work was supported by 10.13039/501100001691JSPS KAKENHI (grant numbers: 22K16036 [N.K.] and 22H03055 [S.T.]).

## Declaration of competing interest

The authors declare the following financial interests/personal relationships which may be considered as potential competing interests: Naruhiro Kimura reports financial support was provided by JSPS KAKENHI Grant. If there are other authors, they declare that they have no known competing financial interests or personal relationships that could have appeared to influence the work reported in this paper.

## Figures and Tables

**figs1:**