# Supplementary material for: Mesenchymal stromal cell administration promotes macrophage-mediated bile duct regeneration
Source: Regen Ther. 2026 Feb 3;31:101065. doi: 10.1016/j.reth.2026.101065 (PMC12891882; doi:10.1016/j.reth.2026.101065)
Supplement: Multimedia component 1 [file mmc1.docx]

Supplemental Table 1

| antibody | catalog number | dilution |
| --- | --- | --- |
| CK19 (Troma) | MABT913 | 1:500 |
| ki67 | ab15580 | 1:200 |
| ki67 | 14-5698-82 | 1:200 |
| Ck19 | ab7754 | 1:200 |
| Hnf4α | PA5-142255 | 1:500 |
| Sox9 | ab185230 | 1:200 |
