# Supplementary material for: Mesenchymal stromal cell administration promotes macrophage-mediated bile duct regeneration
Source: Regen Ther. 2026 Feb 3;31:101065. doi: 10.1016/j.reth.2026.101065 (PMC12891882; doi:10.1016/j.reth.2026.101065)
Supplement: Multimedia component 2 [file mmc2.docx]

Supplemental Table 2

| antibody | Clone | Cat number |
| --- | --- | --- |
| Zombie NIR |  | 423106 |
| CD45 | 30-F11 | 103129 |
| F4/80 | BM8 | 123147 |
| CD11b | M1/70 | 101237 |
| Ly6c | HK1.4 | 128031 |
